# Trabecular Bone Score and Bone Quality in Systemic Lupus Erythematosus Patients

**DOI:** 10.3389/fmed.2020.574842

**Published:** 2020-09-30

**Authors:** Barbara Ruaro, Andrea Casabella, Sabrina Paolino, Elisa Alessandri, Massimo Patané, Emanuele Gotelli, Alberto Sulli, Maurizio Cutolo

**Affiliations:** ^1^Pulmonology Department, University Hospital of Trieste, Trieste, Italy; ^2^Research Laboratory and Academic Division of Clinical Rheumatology, Department of Internal Medicine (Di.M.I.), IRCCS San Martino Polyclinic Hospital, University of Genova, Genoa, Italy; ^3^Osteoporosis, Bone and Joint Disease Research Center, CROPO, Department of Internal Medicine Di.M.I., University of Genoa, Genoa, Italy; ^4^Lupus Clinic, Department of Internal Medicine (Di.M.I.), IRCCS San Martino Polyclinic Hospital, University of Genova, Genoa, Italy

**Keywords:** osteoporosis, trabecular bone score (TBS), bone mineral density (BMD), autoimmune diseases (AD), bone loss

## Abstract

**Background:** Systemic lupus erythematosus (SLE) patients run a higher risk of having low bone mass due to multifactorial events that include physical inactivity, persistent inflammation, low vitamin D levels, and glucocorticoid treatment. This study aimed at obtaining a comparison between bone involvement in SLE patients and healthy matched subjects (HS).

**Methods:** A total of 40 SLE females (average age 54.1 ± 16.3 years) and 40 age–gender matched HS (average age 54.2 ± 15.9 years) were enrolled after having obtained informed written consent. Bone mineral density (BMD, g/cm^2^) of the lumbar spine (L1–L4) was analyzed by a dual-energy X-ray absorptiometry (DXA) scan (GE, Lunar Prodigy). The lumbar spine trabecular bone score (TBS) was derived for each spine DXA examination by the TBS index (TBS iNsight Medimaps).

**Results:** The lumbar spine TBS score was statistically significantly lower in SLE patients than in HS (0.797 ± 0.825 vs. 1.398 ± 0.207, *p* < 0.001, as was BMD (*p* < 0.001) in all areas examined.

**Conclusions:** SLE is associated with significant low bone mass as evidenced by DXA and TBS. This study emphasizes the importance of using DXA and TBS in the evaluation of the different aspects of bone architecture.

## Highlights

- TBS is a diagnostic tool for the quantification of the bone quality in rheumatic diseases.
- Systemic lupus erythematosus (SLE) patients had a high trabecular bone loss.
- SLE patients have an increased risk of lower bone mass than healthy subjects.

## Introduction

Systemic lupus erythematosus (SLE) is one of the most complex multisystemic autoimmune diseases as it encompasses a wide spectrum of clinical and serological manifestations (1, 2). Recent studies demonstrate a higher incidence of osteoporosis (OP) and fractures in SLE patients than in healthy subjects (HS) (3–5). There is a multifactorial etiology of bone loss in SLE, which includes systemic inflammation; serological, metabolic, and hormonal factors; and maybe also genetic factors and medication (5–8). Moreover, a high prevalence of morphometric vertebral fractures was observed in SLE patients, although 1/3 had normal bone density, in line with the multifactorial etiology of fractures in SLE (3–5).

The clinical consequences and economic burden of OP and fractures highlight the importance of a not only correct, but also early diagnosis of OP so the most suitable preventive therapies can be started.

Several studies demonstrate that patients with rheumatic inflammatory diseases have low vitamin D and an increased risk of low bone mass (3, 6–8). Currently, the clinical practice gold standard for OP diagnosis, including secondary causes, is a bone mineral density (BMD) analysis by dual-energy X- ray absorptiometry (DXA) (9–11).

It has recently been reported that the trabecular bone score (TBS), an index extracted by DXA that provides an indirect measurement of bone axial microarchitecture, provides vital information on bone quality in several rheumatic diseases (6, 7, 12).

The aims of this study were to assess bone involvement in SLE and to compare the results with matched HS using TBS and DXA.

## Methods

### Study Population

A total of 40 female patients affected by SLE (2012 criteria) (13) (average age 54.1 ± 16.3 SD years, average disease duration 5.2 ± 4.9 years) and 40 age–gender matched HS (average age 54.2 ± 15.9 years) were enrolled after obtaining written informed consent during routine clinical assessment in our rheumatology department from January 2015 to November 2017. All SLE and HS were in the postmenopausal period (see Table 1).

**Table 1:** Clinical findings in patients with systemic lupus erythematosus (SLE) and healthy subject (HS), prednisolone (PRED, average 5 mg/day), hydroxychloroquine (HCQ, average 150 mg/day), mycophenolate (MMF, average 1,500 mg/day), azathioprine (AZA, average 50 mg/day), proton pump inhibitors (PPI), Not Applicable (NA).

	**SLE#40**	**HS#40**	***p* value**
	**Median (IQR)**	**Median (IQR)**	
Age (years)	55 (12)	54 (11)	*p* > 0.05
Age of menopause (years)	49 (6)	50 (5)	*p* > 0.05
Gender (Female/Male)	40/0	40/0	NA
BMI (kg/m^2^)	22 (2)	23 (3)	*p* > 0.05
Disease duration (years)	6 (5)	NA	NA
Autoantibodies (dsDNA/negative)	34/6	0/40	NA
SLEDAI	5 (2)	NA	NA
Smoking status, current (number)	3/40	4/40	*p* > 0.05
25(OH)D (ng/ml)	15 (4)	28 (2)	*p* < 0.001
ALP bone (UI)	7 (2)	17 (3)	p < 0.001
Treatments (PRED/MMF/HCQ/AZA/PPI)	21/8/20/6/2	0/0/0/0/2	NA

A complete medical history was collected and a clinical examination performed for all subjects enrolled. Demographic data, such as age, gender, height, weight, and body mass index (BMI) were also recorded (see Table 1).

All patients were not affected by secondary causes of OP, such as metabolic or endocrinological disease or drug-induced OP.

The inclusion criteria were an SLE diagnosis and having been on a stable drug regimen for at least 4 months prior to study entry.

The exclusion criteria were being on a drug regimen that could potentially influence the final data, such as vitamin D and/or bisphosphonate, and a glucocorticoid dosage of more than 7.5 mg/day in the 4 months prior to study entry.

Any concomitant treatment in SLE patients and in HS are reported in Table 1.

The patients' history included information as to vertebral and nonvertebral fractures assessed by lateral spinal radiographs of the thoracic and lumbar spine and other districts whenever possible.

### Bone Mineral Density

The BMD of the lumbar spine (L1–L4) and left hip (femoral neck; Ward's triangle; trochanter; total hip) was obtained by a DXA scan (Lunar Prodigy, GE Lunar, Madison, WI, USA) in both groups. The weight, height, age, and gender of each subject were used to estimate the BMD, expressed as a T-score (expressed as g/cm^2^ and standard deviation scores) and compared to the HS BMD values. This number shows the amount of bone present compared to a young adult of the same gender with peak bone mass (9–11). A score above−1 is considered normal in BMD T-scores obtained at the femur and lumbar spine, a score between−1 and−2.5 is classified as osteopenia (low bone mass), and a score below−2.5 is classified as OP.

We also calculated the Z-score. This number reflects the amount of bone present compared to others in the same age group of the same size and gender. If this score is unusually high or low, further medical tests may be advisable (9–11).

### Trabecular Bone Score

The lumbar spine TBS, a texture analysis parameter correlated to the bone micro-architecture parameters (9, 12), was derived for each spine DXA examination by the TBS index (TBS iNsight Medimaps). The lumbar spine L1–L4 TBS was calculated on each spine DXA examination with the operator blinded to clinical parameters and outcomes (6, 7, 12).

A normal range for TBS values in postmenopausal women has been proposed: a TBS of ≥1.350 is considered normal; a TBS between 1.200 and 1.350 is considered consistent with partially degraded microarchitecture, and a TBS of ≤ 1.200 defines degraded microarchitecture (9, 12).

### Bone Parameters

After obtaining written informed consent, a complete blood chemistry evaluation of bone metabolism was made (alkaline phosphatase, parathormone, 25 OH vitamin D, calcium, and phosphorus) (6, 7) for all subjects enrolled.

### Statistical Analysis

Statistical analyses were performed by Graph Pad PRISM version 5.02. Nonparametric tests were used for the statistical analysis. The Mann–Whitney *U*-test was performed to compare unpaired groups of variables and the Kruskal–Wallis test to compare continuous variables with nominal variables with more than two levels. The Spearman rank correlation test was used to search for any relationships between variables along with linear regression tests. Any *p*-values lower than 0.05 were considered statistically significant. The results are reported as mean along with standard deviation (SD) and median and interquartile range (IQR).

## Results

The lumbar spine TBS score was significantly lower in SLE patients than in HS (0.797 ± 0.825 vs. 1.398 ± 0.207, *p* < 0.001), and BMD was significantly lower in all areas (the lumbar spine, femoral neck, Ward's triangle, trochanter, and hip) than in the HS group (*p* < 0.001 for all areas) (See Table 2). There was a 39% and 16% prevalence of osteopenia and OP in SLE patients, respectively. Most SLE patients (60%) had a significantly lower bone loss than the HS group (*p* < 0.001). A total of 30% of the SLE patients had a history of high-dose oral glucocorticoids in the 2 years before the study (> 10 mg/day), and this was associated with the preservation of BMD in the lumbar spine but not in the spinal trabecular bone as observed in the TBS analysis.

**Table 2:** Trabecular bone score (TBS) and bone mineral density (BMD) values in systemic lupus erythematosus (SLE) patients and controls (CNT).

	**SLE, *n* = 40 median (IQR)**	**HS, *n* = 40 median (IQR)**	***p* value**
TBS	0.803 (0.729)	1.344 (0.236)	*p* < 0.001
Lumbar spine (L1-L4) BMD (g/cm^2^)	0.536 (0.392)	1.478 (0.831)	*p* < 0.001
Femoral neck BMD (g/cm^2^)	0.841 (0.352)	0.957 (0.313)	*p* = 0.02
Ward's triangle BMD (g/cm^2^)	0.569 (0.210)	0.736 (0.245)	*p* < 0.05
Trochanter BMD (g/cm^2^)	0.712 (0.206)	0.895 (0.216)	*p* = 0.05
Total hip BMD (g/cm^2^)	0.628 (0.572)	1.251 (0.314)	*p* = 0.01

A total of 24 SLE patients (40%) had a previous vertebral fracture, and all the patients with previous vertebral fractures had a low bone mass, 29 patients (42%) had osteoporosis, and 32 (54%) osteopenia.

Noteworthy is the fact that the TBS values in the SLE group had a positive correlation with the BMD values measured at the level of the spine (*p* = 0.04), femoral neck, and the whole femur (*p* < 0.01, respectively).

SLE patients had statistically significantly lower serum levels, i.e., 25 (OH) D, than did the HS (17.1 ± 2.3 ng/ml vs. 27.8 ± 1.4 ng/ml, *p* < 0.001).

A total of 70% of the SLE patients had a 25 (OH) D insufficiency (<30 ng/ml) as did 10% of the HS.

SLE patients with previous fractures had statistically significantly lower vitamin D values than those without vertebral fractures (7.4 ± 6.6 ng/ml vs. 15.2 ± 7.6 ng/ml; *p* < 0.0001). Bone alkaline phosphatase (ALP bone) level was lower in the SLE patients than in the HS (7.5 ± 2.1U/l vs. 18.5 ± 4.5 U/l; *p* < 0.001). No statistically significant differences were observed between SLE and HS groups for serum calcium, phosphorus, or PTH.

In addition, no differences regarding the most important risk factors for OP, such as smoking condition, alcohol consumption, age of menopause, and familiarity with hip fractures, were observed between SLE patients and the healthy subjects.

There was no correlation between the TBS values and the BMD measured at the spine, femoral neck, and level of the whole femur or between the vitamin D, PTH, calcium, and phosphorus values in SLE patients and HS. Although there was a positive correlation between the TBS values and the bone alkaline phosphatase values in the SLE patients (*p* = 0.01), no statistically significant correlation was observed between the TBS values and SLE disease duration in years or the SLEDAI value.

## Discussion

To the best of our knowledge, this is the first study to assess bone involvement in SLE and to compare the results with matched HS using TBS and DXA.

This study confirms that SLE is associated with significant trabecular bone loss. This study emphasizes the pivotal role TBS plays as an innovative and safe diagnostic tool for the quantification of bone quality in chronic and systemic inflammatory rheumatic diseases, such as SLE.

Osteoporosis is the most serious bone metabolic disorder and is characterized by a reduction of bone mass and micro-architectural deterioration associated with an increased risk for fragility fractures (1–3). As it frequently involves patients with rheumatic diseases, the high morbidity and mortality of fractured subjects and the increased socioeconomic costs suggest it can be considered a major health problem. Therefore, there is a need for either a precocious identification of subjects with fragile “bones” or the institution of specific diagnostic–therapeutic strategies. Enhanced knowledge of bone pathophysiology coupled with progress in pharmaceutical development has provided the opportunity to make early identification of subjects at high risk of fragility fractures and to start preventive therapy (13, 14).

This study also confirms that patients with SLE, a complex systemic autoimmune connective tissue disease, have an increased risk of bone loss (osteopenia and OP) and fractures (15, 16). The increased risk of bone loss associated with SLE is multifactorial and caused by a lack of motor activity, disturbance of hormonal balance, increased inflammatory cytokines, kidney impairment, nutritional disorders, vitamin D deficiency, and medications such as corticosteroids (17–20). In particular, long-term use of corticosteroids may induce OP in patients with SLE by influencing their bone turnover, increasing bone resorption, and decreasing bone formation, preventing the formation of collagen and osteocalcin as well as reducing bone matrix mineralization (17–20). In addition, there are many cytokines involved in the pathogenesis of OP. Recently, several studies underline the important role in the osteoporotic process of IL-33 (21–23). These studies indicate that IL-33 may play a role in bone remodeling, likely influencing osteoblast and osteoclast function. Furthermore, IL-33 is an inducer of Th2 immune responses and presents a pivotal role in the development of many autoimmune diseases, such as SLE (21–23).

The data of our study also confirm that SLE patients have an increased risk of 25 (OH) D insufficiency as frequently reported in rheumatic diseases (24, 25). The effect of vitamin D on improving the BMD in SLE patients is yet unclear. However, vitamin D regulates several genes involved in innate and adaptive immunity, so it can possibly play a role in SLE through its immunomodulatory effects, which include downregulating Th1 immune responses, modulating the differentiation of dendritic cells, reducing the proliferation of activated B-cells, upregulating regulatory T-cells, and preserving innate immune responses (24, 25). Further, vitamin D has also been found to hinder the production of interferon alpha, which is known to play a key role in the etiology and pathogenesis of SLE (24, 25). Recent studies have shown that long-term supplementation with different doses of vitamin D (400–1200 IU) and calcium (1–1.5 g) can have a better effect on the BMD of postmenopausal osteoporotic women (25–27). A recent study indicates that supplementation with higher doses of vitamin D (1400 IU cholecalciferol per day) and calcium carbonate (1,250 mg per day) for 6 months improves bone mineral density and decreases the rates of osteopenia and OP in corticosteroid-treated patients. Probably, 1.25 (OH)-D can activate osteoblast and bone formation as well as decrease bone resorption through inactivation of osteoclasts (25–27). Furthermore, the authors underline that high calcium supplementation may improve the bone matrix and, consequently, prevent its destruction (25–27).

Confirming the pivotal role TBS plays in the assessment of bone microarchitecture further, two recent reviews reports a high prevalence of morphometric vertebral fractures although there is normal bone density in 1/3 SLE patients (9, 14–16).

Our present study has some limitations, including (a) a small number of participants, which limits the statistical investigations (e.g., multivariate analysis assessing the possible effect of confounding factors); (b) single-center recruitment; and (c) enrollment of SLE patients with quiescent disease (to reduce the use of treatment that could have created a bias), which could explain the lack of correlation between the TBS values and disease duration or the disease activity index (SLEDAI) in SLE patients.

In conclusion, this study demonstrates that SLE patients run a higher risk of low bone mass and that it is important to evaluate the different aspects of bone architecture with DXA and TBS and bone parameters, such as vitamin D, as soon as possible.

## Data Availability Statement

The raw data supporting the conclusions of this article will be made available by the authors, without undue reservation.

## Ethics Statement

The studies involving human participants were reviewed and approved by the Ethical Committee of San Martino Polyclinic Hospital, Genoa, Italy, and this study has been performed in accordance with the ethical standards laid down in the 1964 Declaration of Helsinki and its later amendments. The patients/participants provided their written informed consent to participate in this study.

## Author Contributions

BR, AC, and MC: substantial contributions to the conception and design of the work, the acquisition, analysis and interpretation of data, drafting the work and revising it critically for important intellectual content, and final approval of the version to be published. SP, EA, MP, EG, and AS: substantial contributions to the analysis, interpretation of data, final approval of the version to be published. All authors contributed to the article and approved the submitted version.

## Conflict of Interest

The authors declare that the research was conducted in the absence of any commercial or financial relationships that could be construed as a potential conflict of interest.
